# Clinical profile of children with influenza like illness during pre-monsoon at coastal Karaikal, Puducherry, India

**DOI:** 10.6026/973206300200252

**Published:** 2024-03-31

**Authors:** Dande Naga Mahesh, B Sreelatha, S Vinoth, S Nancy

**Affiliations:** 1 Department of Paediatrics, Vinayaka Mission's Medical College and Hospital, Vinayaka Mission's Research Foundation - Deemed to be University (VMRF-DU), Karaikal, Puducherry, India; 2 Department of Community Medicine, Vinayaka Mission's Medical College and Hospital, Vinayaka Mission's Research Foundation - Deemed to be University (VMRF-DU), Karaikal, Puducherry, India

**Keywords:** Covid-19, CRP, ESR, influenza like illness (ili), cross-sectional study

## Abstract

Influenza infections in developing countries are under reported and WHO estimates that nearly 99% of influenza deaths worldwide occur in children under-five years of age in Asian and African countries. Consequently, this study aims to analyze the use of clinical profile and easily available laboratory parameters to aid identification of the possible viral etiology in the setting of pre-monsoon ILI. A cross-sectional study was carried out for three months among children with ILI attending fever clinic of a tertiary care hospital in Karaikal, South India. In the study population the prevalence of ILI was highest in the age group four to five years followed by school aged children. Adolescents were affected the least. Influenza B was most common virus causing ILI in this region, followed by covid-19 infection. Laboratory parameters depicted a significantly high ESR in COVID-19 infected ILI children. They also exhibited leucopenia and normal platelet counts. Clinical symptoms and laboratory parameters which are easily available and cheaper can be used in resource poor settings of healthcare to identify possible influenza and COVID-19 infected children amongst cases presenting with ILI.

## Background:

Influenza like illness (ILI) is defined as constitutional symptoms including fever (temperature more than 100 O F), cough and sore throat [1]. This category of clinical presentation is used worldwide for influenza surveillance [2]. The most important viruses causing such clinical picture are influenza viruses and covid-19 infections [3]. ILI affects 9 - 49 million people worldwide every year, most of which are reported from western world [1]. In tropical countries, influenza occurs throughout the year causing outbreaks more irregularly [1,2]. Influenza infections in developing countries are under reported and WHO estimates that nearly 99% of influenza deaths worldwide occur in children under 5 years of age in Asian and African countries [2]. The common viruses causing clinical picture of fever, cough and sore throat include Rhinovirus, Respiratory Syncytial Virus, Influenza viruses (mostly A and B, rarely C), Corona virus, Adenovirus, SARS Severe Acute Respiratory Syndrome (SARS) virus, MERS (Middle East Respiratory Syndrome) virus [3]. Of these, COVID-19 pandemic ran havoc for nearly two years. It was managed effectively with clinical triage; standard rapid diagnostic kit based viral antigen detection, risk categorization and standard treatment protocols [4]. Early development of herd immunity with the help of mass vaccination and to a lesser extent infection derived immunity helped to curtail the pandemic [4]. But more recurrent and persistent are influenza and ILI causing significant mortality and morbidity among high-risk category of adults and children less than 60 months in the community [5]. This is more difficult to control because of its frequent antigenic shift and drift requiring yearly vaccination which is practically difficult in populous and economically backward countries [3]. To an equal extent other less virulent viruses contribute to ILI presentation in children, making viral detection a heavy burden in healthcare infrastructure and manpower [6]. With reopening of schools in post COVID era, hospitals started facing high burden of flu like symptoms in children because of immune debt and humid climate [7]. In the context of poor resource healthcare setup and the need to identify possible viruses causing ILI among children especially among the high-risk pediatric population, the present study aims to analyze the use of clinical profile and easily available laboratory parameters to aid identification of the possible viral etiology in the setting of pre-monsoon influenza like illness (ILI) [5]. This would also help in prediction of progression to severe illness in children presenting initially with ILI, early initiation of appropriate antiviral drugs in high-risk category and at the same time avoid their inadvertent use [7]. Epidemiological importance of identifying viral etiology in ILI is also highlighted in the study which may help in diagnostics, mitigation, modeling and preparation of future pandemics [8]. Therefore, it is of interest to analyze the use of clinical profile and easily available laboratory parameters to aid identification of the possible viral etiology in the setting of pre-monsoon influenza like illness (ILI).

## Methodology:

A cross-sectional study was done among children attending fever clinic of a tertiary care hospital in Karaikal, South India (between June 1, 2022 and August 31, 2022). All children with ILI attending the fever clinic were enrolled in the study. Children with asthma, congenital heart diseases, and chronic diseases and on long term drugs were excluded from the study. A total of 148 children between 4 to 17 years were included in the study. Of this, parents of 113 children gave consent for complete evaluation including laboratory investigations. Clinical evaluation including detailed history, contact history, general physical examination, anthropometry, vital signs measurement was carried out. Thorough examination of respiratory system was documented. Other systems were examined to identify multi system involvement. Subsequently, children were subjected to analysis of laboratory parameters including complete blood count, inflammatory markers like CRP using Horiba 3- part fully automatic CBC, CRP Analyzer, ESR (Wintrobe tube), liver enzymes (Indiko Plus Biochemistry Analyzer). Nasopharyngeal and oropharyngeal swab for viral PCR, study panel for Influenza A and B, Adenovirus, Boca virus, H1N1, H3N2, RSV and Rhinovirus were done (Filmarray Respiratory Panel 1 Biomerieux, India). The results were statistically analyzed using Epi -info package computed with SPSS - version 24 software. Categorical variables were expressed as frequencies and percentages and the differences between two categorical variables were determined using chi-square test. P value < 0.05 was considered statistically significant difference.

## Ethical consideration:

The study was carried out after obtaining approval from the Research Committee and Institutional Ethics Committee (EC approval number: 43/2022).

## Results:

## Age and gender distribution of study population:

**Figure 1:**
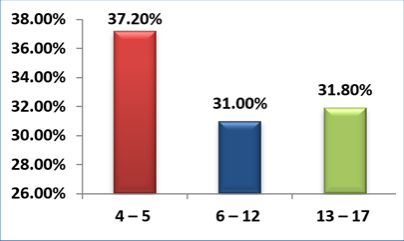
Age distribution [N = 113]

**Figure 2:**
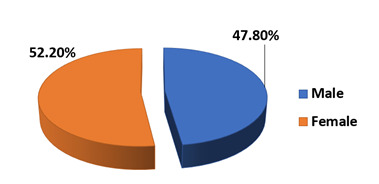
Gender distribution [N=113]

Among 113 children, 98 tested positive for a single virus by PCR. 7 tested positive for more than one virus in the same panel of PCR and were excluded from analysis. The study population was divided into three groups based on age as preschool age (4-5 years), school age (6-12 years) and adolescent age (13-17 years) which were comparable in number. Gender distribution was also comparable among the study population as shown (Figure 1 & Figure 2).

## Cases tested positive for a single virus and types of viruses:

**Figure 3:**
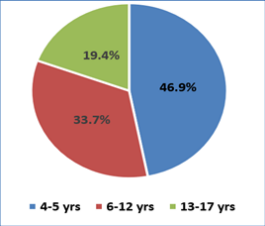
No. of cases tested positive for a single virus [N=98]

**Figure 4:**
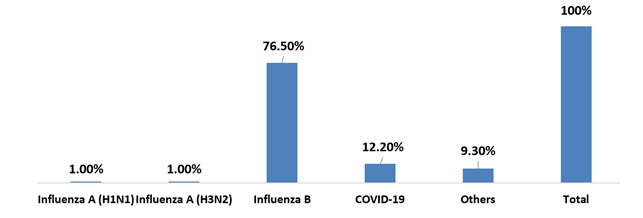
Type of virus [N=98]

In the study population the prevalence of ILI was highest in the age group 4-5 years followed by school aged children. Adolescents were affected the least. Girls were more commonly affected by ILI in the preschool and adolescent age and boys in school age. In the pre-monsoon season, Influenza B was most common virus causing ILI in this region (n=75, 76.5%) followed by covid-19 infection (n=12, 12.2%). Influenza A contributed only to a small percentage (n=2, 2%) of pediatric ILI in the pre-monsoon season. Boca virus was positive in one school aged child (1%), adeno virus in 5 children (1 preschool and 3 school aged and 2 adolescents - 5.1%) and rhino virus in 1 child (preschool age, 1%) as shown (Figure 3 & Figure 4).

## Constitutional symptoms and prostration:

**Figure 5:**
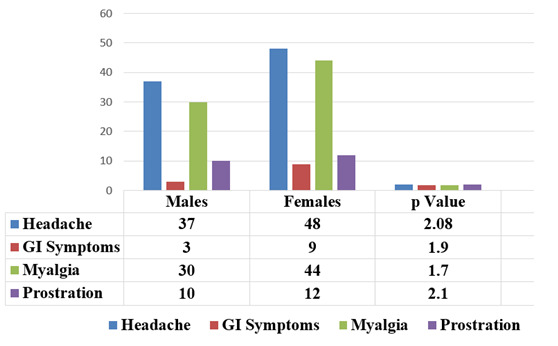
Gender Comparison of Constitutional Symptoms

**Figure 6:**
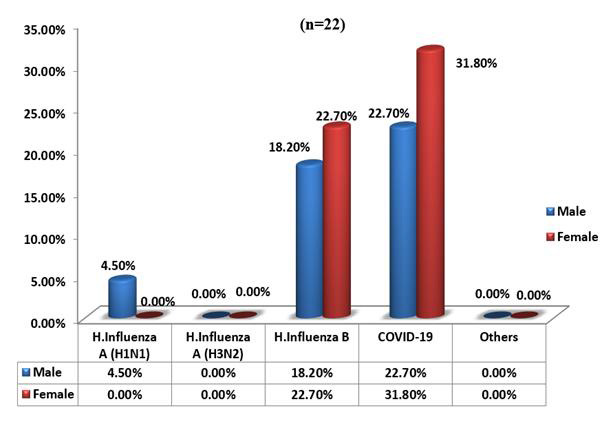
Prostration with comparison of gender and type of virus [N=22]

Clinical manifestations beyond the triad of ILI like headache, chills, toxic general appearance, dehydration, gastrointestinal symptoms, myalgia, and prostration were not significantly different in either sex. Gastro intestinal symptoms of vomiting, diarrhea, dehydration and chills were significantly higher in children with Influenza B and H3N2 viral infections. Prostration was significantly characteristic of children with influenza B and COVID-19 infections (Figure 5 & Figure 6).

## Laboratory parameters:

In the present study, laboratory parameters depicted a significantly high ESR in COVID-19 infected ILI children. They also exhibited leucopenia and normal platelet counts. High hematocrit, leukocytosis, thrombocytosis, low mean platelet volume was significantly more common in Influenza B and thrombocytopenia in influenza A and COVID-19 infections in this study. In the present study, H3N2 infection has significantly high mean platelet volume (MPV) to platelet ratio. CRP is an inflammatory marker and doesn't show any significant variation with different virus species causing ILI. In the present study, there was no progression to severe disease in any child that CRP did not show statistically significant variation among various age, sex or viral types causing the illness (Table 1).

## Discussion:

In the study population, the prevalence of ILI was highest in the age group 4-5 years followed by school aged children. Adolescents were affected the least. Girls were more commonly affected by ILI in the preschool and adolescent age and boys in school age. In the pre-monsoon season, Influenza B was most common virus causing ILI in this region (n=75, 76.5%) followed by covid-19 infection (n=12, 12.2%). Influenza A contributed only to a small percentage (n=2, 2%) of pediatric ILI in the pre-monsoon season. Boca virus was positive in one school aged child (1%), adeno virus in 5 children (1 preschool and 3 school aged and 2 adolescents - 5.1%) and rhino virus in 1 child (preschool age, 1%). Notably, this is comparable to a pre covid Japanese study by Mitsuo *et al.* which revealed 70.2% of ILI in school aged children in their season of influenza [9]. The present study also depicts that, 78.5% cases of ILI are due to influenza viruses. The strain is Influenza B in the present study whereas viral typing was not conducted in the Japanese study. The age group in the present study is wide in contrast to the Mitsuo study where only 6-12year children were enrolled. Even in the post COVID-19 pandemic era, Influenza virus dominated the seasonal ILI. This is in contrast to an Australian study by Taylor *et al.* [10]. The age group of the study was 6 months to 10 years and it was in the monsoon season when 15.8% was due to influenza viruses, 9.8% was caused by adeno virus, RSV 9.7%, corona virus 5.6%, human meta-pneumo-virus 5.5% and Boca virus 2%. The sample size was high 6626 but adolescents were not included [10].

Clinical manifestations beyond the triad of ILI like headache, chills, toxic general appearance, dehydration, gastrointestinal symptoms, myalgia, and prostration were not significantly different in either sex. Gastro intestinal symptoms of vomiting, diarrhea, dehydration and chills were significantly higher in children with Influenza B and H3N2 viral infections. This is in line with a study by Charisma *et al.*, which describes significantly higher gastro intestinal symptoms among influenza infected children of Indonesia. Influenza B is more commonly identified in children with gastrointestinal symptoms than influenza A. Influenza viruses bind to α2,6 sialic acid receptors in gastrointestinal tract, the expression of which are more in larger surface area of pediatric jejunum and ileum [11] causing gastrointestinal symptoms. Dehydration in influenza occurs due to several reasons including higher water loss through the greater surface area of child's skin during high fever of influenza infection, poor intake of oral fluids by affected children, concomitant vomiting and diarrhea [12]. Chills in children with Influenza are attributable to higher inflammatory response characterized by higher levels of IL-1 and TNF α. This is a marker of high viral load, low pre-existing individual immunity due to immune debt and poor coverage of influenza vaccination in India. In our study, chills are predominantly seen in children with Influenza B infection. This is in contrast to a study by Tsung-Pie-Tsu where Influenza A (H5N1) was found to be more prevalent and chills were related to higher viral load. The prevalence of influenza strains varies between regions and different seasons [13].

Prostration was significantly characteristic of children with influenza B and COVID-19 infections. Similar findings are documented in a Brazilian study where prostration was more prevalent in children infected with Influenza B virus than other viruses. It is one of the manifestations of high circulating levels of inflammatory mediators especially IL-1. This carries a good prognosis as adequate viral elimination occurs [14]. In the present study, laboratory parameters depicted a significantly high ESR in COVID-19 infected ILI children. They also exhibited leucopenia and normal platelet counts. An earlier study by Sowjanya *et al.*, identified lymphopenia in children with Influenza A infection and hypothesized bone marrow suppression as the reason for such suppression [15]. A similar publication by J Rice *et al.*, correlated leukopenia to severity of seasonal Influenza A infection in children in 1998 and associated the progression to pneumonia in cases who had early leukopenia [16].

High hematocrit, leukocytosis, thrombocytosis, low mean platelet volume was significantly more common in Influenza B and thrombocytopenia in influenza A and COVID-19 infections in this study. An article by Vojko Berce *et al.* in 2015 revealed low platelets in H5N1 and high in H1N1 infections in preschool age. But influenza B was not included in the study population. It also demonstrated high white cell count, thrombocytosis, low mean platelet volume in lower respiratory tract infections by adeno virus in the same category [17] Serial increase in mean platelet volume (δ) estimation was used to predict the progression to pneumonia in children and ARDS in adults during Influenza pandemics in Thailand study by Teeraphat *et al.* [18]. It has been identified that platelets engulf viral particles of Influenza which bind to platelet sialoglycans resulting in the removal of sialic acid by viral neuraminidase. These naked platelets with the attached influenza virus are cleared by the liver. The clearance was triggered by the altered platelet antigenic structure. Same theory is applicable to COVID-19 induced thrombocytopenia [19]. High PCV and leukocytosis in the context of thrombocytopenia and low platelet volume goes against pancytopenia, a marker of hemophagocytosis which may complicate any viral infection. In the present study, H3N2 infection has significantly high mean platelet volume (MPV) to platelet ratio. High MPV to platelet ratio has all along been considered as a surrogate marker of Influenza A severity in ILI children. This is attributed to increase in platelet production, shortening of platelet cycle time and destruction of megakaryocyte formation in the bone marrow as described in a Chinese study of Influenza pandemic 2019 [20].

CRP is an inflammatory marker and doesn't show any significant variation with different virus species causing ILI. Usually, CRP is found to be elevated in adeno virus and severe Influenza infections. Our study group children had no severe influenza features with only a few cases of adeno viral infection so that CRP did not show a significant difference [21]. ESR is an inflammatory marker in conditions affecting fibrinogen levels and is more helpful in subacute and chronic infections whereas CRP is more helpful in acute infections and in assessment of response to treatment [22]. Elevated inflammatory markers especially CRP and PCT (platelet crit) have been used as surrogate markers of the severity of respiratory tract involvement like pneumonia in Influenza and covid19 infection [23].

## Conclusion:

In conclusion, clinical symptoms and laboratory parameters which are easily available and cheaper can be used in resource poor settings of healthcare to identify possible influenza and COVID-19 infected children amongst cases presenting with ILI. This will help in isolation, antiviral therapy usage in discretion and prevention of complications in high-risk children. This is more an option where rapid diagnostic tests for viral markers/ antigens are unaffordable or inaccessible. Prostration and chills due to high circulating inflammatory mediators in Influenza B cases though troublesome to children and care givers in fact carry a better prognosis. High leukocyte count, high platelet counts with low mean platelet volume in such cases clearly prove an adequate immune response against viral infection. Low mean platelet volume indicates viral clearance in course of the illness. Increasing MPV to platelet could predict increasing severity in ILI children. These parameters can be studied more elaborately involving larger sample size to form standard guidelines in management of children with ILI.

## Financial support and sponsorship:

None

## Figures and Tables

**Table 1:** Laboratory parameters

**LAB VALUES**	**TOTAL NUMBERS**	**LEUKO PENIA**	**LEUKO CYTOSIS**	**NORMAL WBC COUNT**	**HIGH ESR**	**THROMBO CYTOPENIA**	**THROMBO CYTOSIS**	**HIGH CRP**	**LOW MPV**	**HIGH MPV**	**HIGH MPV/PLT**
VIRUS	N	n	n	n	n	n	n	n	n	n	n
		(p value)	(p value)	(p value)	(p value)	(p value)	(p value)	(p value)	(p value)	(p value)	(p value)
H1N1	1	--	--	1	--	1	--	--	--	--	--
H3N2	1	--	1	--	1	1	--	1	--	1	1
			(p<0.05)		(p<0.05)	(p<0.05)		(p<0.05)		(p<0.05)	(p<0.05)
Influenza B	75	2	70	3	5	1	69	2	65	--	--
		(>1.0)	(p<0.001)	(>1.0)	(>1.0)	(>1.0)	(p<0.001)	(>1.0)	(p<0.001)		
COVID-19	12	5	4	3	8	9	1	1	1	--	--
		(>1.0)	(>1.0)	(>1.0)	(p<0.05)	(p<0.05)	(>1.0)	(>1.0)	(>1.0)		
ADENO VIRUS	5	--	3	2	1	--	4	3	--	--	--
			(p<0.001)	(>1.0)	(>1.0)		(p<0.001)	(p<0.001)			
BOCA VIRUS	1	--	--	1	--	--	--	--	--	--	--
RHINO	1	1	--	--	--	--	--	--	--	--	--
RSV	2	1	--	1	--	--	--	--		--	--
